# Feeding broiler chickens with arginine above recommended levels: effects on growth performance, metabolism, and intestinal microbiota

**DOI:** 10.1186/s40104-023-00839-y

**Published:** 2023-03-03

**Authors:** Giorgio Brugaletta, Marco Zampiga, Luca Laghi, Valentina Indio, Chiara Oliveri, Alessandra De Cesare, Federico Sirri

**Affiliations:** 1 Department of Agricultural and Food Sciences, Alma Mater Studiorum – University of Bologna, Ozzano Dell’Emilia, 40064 Bologna, Italy; 2 Department of Veterinary Medical Sciences, Alma Mater Studiorum – University of Bologna, Ozzano Dell’Emilia, 40064 Bologna, Italy; 3 Department of Physics and Astronomy, Alma Mater Studiorum – University of Bologna, 40127 Bologna, Italy

**Keywords:** Arginine, Broiler chicken, Growth performance, Metabolism, Microbiota

## Abstract

**Background:**

Arginine is an essential amino acid for chickens and feeding diets with arginine beyond the recommended levels has been shown to influence the growth performance of broiler chickens in a positive way. Nonetheless, further research is required to understand how arginine supplementation above the widely adopted dosages affects metabolism and intestinal health of broilers. Therefore, this study was designed to assess the effects of arginine supplementation (i.e., total arginine to total lysine ratio of 1.20 instead of 1.06–1.08 recommended by the breeding company) on growth performance of broiler chickens and to explore its impacts on the hepatic and blood metabolic profiles, as well as on the intestinal microbiota. For this purpose, 630 one-day-old male Ross 308 broiler chicks were assigned to 2 treatments (7 replicates each) fed a control diet or a crystalline *L-*arginine-supplemented diet for 49 d.

**Results:**

Compared to control birds, those supplemented with arginine performed significantly better exhibiting greater final body weight at D49 (3778 vs. 3937 g; *P* < 0.001), higher growth rate (76.15 vs. 79.46 g of body weight gained daily; *P* < 0.001), and lower cumulative feed conversion ratio (1.808 vs. 1.732; *P* < 0.05). Plasma concentrations of arginine, betaine, histidine, and creatine were greater in supplemented birds than in their control counterparts, as were those of creatine, leucine and other essential amino acids at the hepatic level. In contrast, leucine concentration was lower in the caecal content of supplemented birds. Reduced alpha diversity and relative abundance of Firmicutes and Proteobacteria (specifically *Escherichia coli*), as well as increased abundance of Bacteroidetes and *Lactobacillus salivarius* were found in the caecal content of supplemented birds.

**Conclusions:**

The improvement in growth performance corroborates the advantages of supplementing arginine in broiler nutrition. It can be hypothesized that the performance enhancement found in this study is associated with the increased availability of arginine, betaine, histidine, and creatine in plasma and the liver, as well as to the ability of extra dietary arginine to potentially ameliorate intestinal conditions and microbiota of supplemented birds. However, the latter promising property, along with other research questions raised by this study, deserve further investigations.

**Supplementary Information:**

The online version contains supplementary material available at 10.1186/s40104-023-00839-y.

## Background

Arginine is a versatile amino acid that plays proteinogenic, trophic, and functional roles in the animal body [1–3]. Being multifunctional, arginine can affect metabolism, growth, immunity, and health state in several ways [4–7]. For instance, it is the substrate for the biosynthesis of nitric oxide, polyamines, proline, and glutamate [3]. Nitric oxide is involved in many physiological processes, such as the regulation of cardiovascular and renal functions, inhibition of tumor growth, and modulation of the immune response, to name but a few [8, 9]. Polyamines (i.e., putrescine, spermine, and spermidine) have been shown to regulate gene expression and protein synthesis, as well as proliferation, differentiation, and apoptosis of cells [10, 11]. Proline is a key regulator of cellular metabolism and physiology [12], while glutamate is an essential component of glutathione, the potent antioxidant tripeptide [13]. Arginine has also been demonstrated to induce expression and secretion of anabolic hormones, such as insulin, growth hormone (GH), and insulin-like growth factor-1 (IGF-1) [14–17]. Moreover, arginine affects skeletal muscle development through the mechanistic target of rapamycin (mTOR) pathway [18], and is used to generate creatine, an amino acid derivative that is vital for the function and energy homeostasis of muscles [19]. Over the last two decades, there has also been an increasing interest in the effects of arginine on the gastrointestinal tract. Arginine and its derivatives have consistently been shown to possess gut health promoting and re-establishing properties, such as acceleration of mucosal regeneration and recovery from gastroenteric disorders, improvement of epithelial integrity and barrier function, immunomodulation, anti-inflammatory activity, inhibition of enteric pathogens, and restoration of a desirable microbiota [8, 20–29].

With respect to animal nutrition, arginine is commonly considered a semi-essential or conditionally essential amino acid for adult mammals [3, 14, 30], while chickens exclusively rely on dietary arginine to meet their needs [4, 5]. This is because mammals are ureotelic (i.e., urea-excreting) animals that can endogenously produce arginine de novo with enzymes of the urea cycle, whereas avian species are uricotelic (i.e., uric acid-excreting) organisms unable to complete the urea cycle [4, 5]. In the early 1960s, Tamir and Ratner [31] proved that chickens lack carbamoyl phosphate synthase I, which would catalyze ammonia fixation, and have a scarcely active ornithine transcarbamylase that transfers the fixed nitrogen to ornithine in order to generate citrulline, an indispensable intermediate for the urea cycle. However, pioneering studies conducted almost 30 years before had already suggested that arginine is an essential nutrient for chickens [32, 33]. According to the well-known Nutrient Requirements of Poultry published by the NRC [34], broiler chickens should be given diets containing 1.25%, 1.10%, and 1.00% of arginine up to the 3^rd^, from the 3^rd^ to 6^th^, and from the 6^th^ to 8^th^ weeks of age, respectively, with a constant arginine to lysine ratio of 1.04. Although these guidelines have been adequate for a long time, extensive research has demonstrated that arginine requirements of broilers substantially vary depending on diet composition and environmental conditions [5, 6]. It has also been proved that feeding broilers arginine above the recommended levels, such as those released by the NRC [34] or the breeding companies, is beneficial for their health, growth performance, and processing traits [6]. More studies, however, are required to fully comprehend the roles of arginine at the metabolic and intestinal level of broilers. In this regard, Morris [35] claimed that “-omics” technologies can help advance our knowledge of how arginine affects and modulates animal metabolism. Therefore, the main goals of the present investigation were: i) to study the effects of dietary arginine supplementation above recommended levels on growth performance of broilers and ii) to explore the impacts of this nutritional solution on hepatic and blood metabolic profiles, as well as on the intestinal microbiota of broilers with the application of metabolomics and shotgun metagenomic sequencing.

## Methods

### Experimental design, housing, and husbandry conditions

In this study, approved by the Ethical Committee of the University of Bologna (ID: 4387), birds were reared, monitored, and slaughtered in compliance with EU legislation (Dir. 2007/43/EC; Reg. 2009/1099/EC; Dir. 2010/63/EU).

A total of 630 one-day-old male Ross 308 broiler chicks, obtained from the same breeder flock and hatching batch, were supplied by a commercial hatchery and vaccinated against infectious bronchitis virus, Marek’s disease virus, Newcastle and Gumboro diseases, and coccidiosis. Birds were randomly assigned to 2 treatments (7 replicates/treatment of 45 birds each) that were fed a commercial mash basal diet (control – CON) or the same basal diet supplemented with crystalline *L*-arginine (ARG) for the whole grow-out period (0–49 d). The basal diet was formulated to meet the nutrition specifications released by the breeding company [36]. Analysis of the amino acid concentration of the experimental diets was outsourced to an external laboratory (Evonik Industries AG, Hanau, Germany). Table 1 provides the formula and composition of the basal diet according to the 4-phase feeding program used. For every feeding phase, the basal diet was part of a single batch and the sub-batches intended to be given to ARG replicates were supplemented on top with crystalline *L*-arginine (about 1.5 g/kg feed; purity of 98%; BESTAMINO™, CJ BIO, Seul, Korea). The basal diet had a total arginine level of 1.59%, 1.42%, 1.32%, and 1.25%, while ARG diet of 1.75%, 1.57%, 1.47%, and 1.39% in starter, grower I, grower II, and finisher phase, respectively. The total arginine to total lysine ratio of the basal diet ranged between 1.07 and 1.08 and was consistent with the breeding company’s guidelines [36], whereas that of ARG diet was 1.20 throughout the trial.

**Table 1:** Basal diet formula and composition according to the 4-phase feeding program

Ingredient, g/100 g	Feeding phase			
	**Starter** **(0–9 d)**	**Grower I** **(10–21 d)**	**Grower II** **(22–35 d)**	**Finisher** **(36–49 d)**
Corn	37.60	44.88	34.99	25.00
White corn	0.00	0.00	5.00	5.00
Wheat	12.49	12.51	15.83	22.29
Sorghum	0.00	0.00	5.00	10.00
Pea	3.00	3.00	3.00	3.00
Soybean meal	25.04	17.10	13.26	11.36
Roasted soybean	10.00	15.00	15.00	15.00
Sunflower meal	2.00	2.00	2.00	2.00
Corn gluten meal	3.00	0.00	0.00	0.00
Soybean oil	2.36	2.01	2.69	3.47
Calcium carbonate	0.32	0.56	0.73	0.93
Dicalcium phosphate	1.48	0.54	0.29	0.00
Sodium bicarbonate	0.06	0.07	0.15	0.15
Sodium chloride	0.36	0.31	0.25	0.25
Choline chloride	0.10	0.10	0.05	0.06
Lysine sulphate	0.58	0.56	0.54	0.49
*DL*-methionine	0.31	0.13	0.10	0.05
Methionine hydroxy analogue	0.00	0.24	0.24	0.28
*L*-threonine	0.21	0.17	0.14	0.12
Amino acid mix (arginine, valine, isoleucine)	0.20	0.20	0.19	0.13
Non-starch polysaccharides-degrading enzyme	0.05	0.05	0.05	0.05
Phytase	0.08	0.08	0.08	0.08
Emulsifier	0.05	0.05	0.05	0.05
Mycotoxin binder	0.10	0.00	0.00	0.00
Vitamin and mineral premix^a^	0.50	0.45	0.36	0.25
Calculated and analyzed composition				
Dry matter, %	88.50	88.42	88.30	88.30
Crude protein, %	24.02	20.78	19.52	19.13
Total lipid, %	6.05	6.73	7.47	8.12
Crude fiber, %	2.90	2.91	2.84	2.84
Ash, %	5.31	4.48	4.22	4.07
Total lysine, %	1.47	1.31	1.23	1.16
Total arginine, %	1.59	1.42	1.32	1.25
Total arginine:total lysine^b^	1.08	1.08	1.07	1.08
Total methionine + total cysteine, %	1.10	0.99	0.93	0.89
Calcium, %^b^	0.76	0.60	0.57	0.55
Phosphorus, %^b^	0.65	0.47	0.41	0.36
Metabolizable energy, kcal/kg^b^	3,050	3,152	3,225	3,275

^a^The premix provides the following per kg of feed: vitamin A (retinyl acetate), 12,500 IU; vitamin D_3_, 5000 IU (i.e., cholecalciferol, 3500 IU + 25-OH D_3_, 1500 IU); vitamin E (*DL*-α-tocopheryl acetate), 125 mg; vitamin K (menadione sodium bisulfite), 6.75 mg; riboflavin, 9.0 mg; pantothenic acid, 22.0 mg; niacin, 75 mg; pyridoxine, 5 mg; folic acid, 3.0 mg; biotin, 0.35 mg; thiamine, 4.0 mg; vitamin B_12_, 50 μg; Mn, 100 mg; Zn, 102 mg; Fe, 30 mg; Cu, 15 mg; I, 2.0 mg; Se, 0.35 mg

^b^Calculated values

Replicates were assigned to 14 floor pens (7.6 m^2^/pen) arranged in a block design and equipped with chopped straw as bedding material, two feeders, and nipple drinkers. Birds had ad libitum access to feed and water. At each feeding phase switch, feed residuals were weighed, while feeders were cleaned and refilled. The environmental temperature was modified according to the flock age and the breeding company’s instructions. The artificial photoperiod was of 23L:1D during the first 7 and last 3 d, while of 18L:6D for the remainder days following EU legislation (i.e., Dir. 2007/43/EC) and the breeding company’s guidelines for lightning and pre-processing management.

### Growth performance measurement

On a replicate basis, the number and body weight (BW) of birds were recorded at placement (D0), feeding phase switches (D10/22/36), and slaughter (D49), while feed intake (FI) was measured for each feeding phase. Daily weight gain (DWG), daily feed intake (DFI), and feed conversion ratio (FCR) were calculated for the abovementioned feeding phases separately. Additionally, cumulative growth performance were calculated for the entire rearing period (0–49 d). The number and BW of dead or culled birds were recorded daily to compute the mortality rate and correct performance data for mortality.

### Processing yields and breast muscle myopathies evaluation

Birds were processed in a commercial slaughterhouse at D49 and, on a treatment basis, carcass and cut-up yields were measured according to standard commercial procedures. Breast muscle myopathies, namely white striping (WS), woody breast (WB), and spaghetti meat (SM), were assessed approximately 24 h post-mortem – after chilling, deboning, and skin removal – on a randomly selected sample of breast fillets (*n* = 292 and 288 for CON and ARG, respectively) by means of a 3-point-scale: score 0, normal; score 1, mild myopathy; score 2, severe myopathy [37].

### Sample collection

At the slaughterhouse (D49), 2 birds per replicate (i.e., 14 birds/treatment) were selected – according to the average BW of the specific experimental group – and used for sampling blood, liver, and caecal content. Blood was collected into lithium-heparin vials, kept at room temperature, and centrifuged to get plasma. Plasma was poured into 1.5 mL sterile tubes and stored at −80 °C until metabolomics analysis with proton nuclear magnetic resonance (^1^H-NMR). Hepatic tissue (~ 1 cm^3^) was dissected from the right lobe of the liver, put into 5 mL sterile tubes, frozen in liquid nitrogen, and stored at −80 °C until ^1^H-NMR analysis. Caecal samples, composed of the content of both caeca, were collected in duplicate within 1.5 mL sterile tubes, frozen in liquid nitrogen, and kept at −80 °C until ^1^H-NMR analysis and DNA extraction for shotgun metagenomic sequencing.

### Metabolomics (^1^H-NMR) analysis

Metabolomics analysis of plasma, liver and caecal content was carried out as previously described [38]. Briefly, an ^1^H-NMR solution with D_2_O, containing 3-(trimethylsilyl)-propionic-2,2,3,3-d4 acid sodium salt (TSP) 10 mmol/L and NaN_3_ 2 mmol/L was created. Phosphate buffer 1 mol/L was used to achieve a pH of 7.00 ± 0.02, while TSP was used as a reference for NMR chemical-shift and NaN_3_ avoided bacterial proliferation. Plasma samples were centrifuged (18,630 × *g*; 900 s; 4 °C) and 0.7 mL of supernatant were mixed with 0.1 mL of the ^1^H-NMR solution. Then plasma samples were centrifuged again at the aforementioned conditions. Approximately 0.5 g of each liver sample were homogenized at 14,000 r/min for 20 s with 3 mL of a water solution of trichloroacetic acid (TCA) 7% (w/w), by means of an Ultra-Turrax (IKA, Germany) homogenizer. The so obtained mixtures were centrifuged at the mentioned conditions and 0.7 mL of supernatant were mixed with 0.1 mL of the ^1^H-NMR solution. The pH was further adjusted to 7.00 ± 0.02 with drops of NaOH 9 mol/L and 1 mol/L as needed, prior to a final centrifugation. Caecal content samples (approximately 80 mg) were mixed with 1 mL of bi-distilled water, centrifuged, and processed like the plasma samples.

The ^1^H-NMR spectra were registered (600.13 MHz; 298 K) with an AVANCE™ III spectrometer (Bruker, Milan, Italy) equipped with Topspin v3.5 software. The signals from broad resonances due to large molecules were suppressed with CPMG-filter (400 echoes with a τ of 400 µs and a 180° pulse of 24 µs, for a total filter of 330 ms), while the residual signal of water was suppressed by means of presaturation. This was done employing the *cpmgpr1d* sequence, part of the standard pulse sequence library. Each spectrum was acquired summing up 256 transients constituted by 32,000 data points encompassing a window of 7184 Hz, separated by a relaxation delay of 5 s. The ^1^H-NMR spectra were phase-adjusted in Topspin v3.5 and then exported to ASCII format by means of the built-in script *convbin2asc*. Spectra were processed with R [39] through home-made scripts. Signal assignment was performed comparing their chemical shift and multiplicity with Human Metabolome Database [40] and Chenomx software library (Chenomx Inc., Edmonton, Canada, v10), by means of Chenomx software routines. For each matrix, the absolute concentration of molecules was performed in the sample with the median water dilution, assessed via probabilistic quotient normalization [41]. For the purpose, TSP was used as an internal standard. Differences in water content between samples from the same matrix were considered through probabilistic quotient normalization. The concentration of each molecule was obtained from the area of one of its signals, calculated by GSD (global spectra deconvolution) algorithm implemented in MestReNova software v14.2.0–26256 (Mestrelab research S.L., Santiago De Compostela, Spain), by considering an LOQ (limit of quantification) of 5. This was done after applying a baseline adjustment by Whittaker Smoother procedure and a line broadening of 0.3.

### DNA extraction, shotgun metagenomic sequencing, and bioinformatics analysis

The DNA was extracted from caecal content samples adopting a bead-beating procedure and using the QIAmp® DNA Stool Mini Kit (Qiagen, Milan, Italy) and processed as previously detailed [42]. Total DNA was fragmented and tagged with sequencing indexes and adapters using the Nextera XT DNA Library Preparation Kit (Illumina, San Diego, CA, USA). Shotgun metagenomic sequencing was performed with NextSeq500 (Illumina) 2 × 149 bp in paired-end mode generating a total of 152.5 GB, corresponding to an average of 37.9 Mreads per sample. Filtering of low-quality reads and sequence adapters trimming of raw reads were conducted using the tool *AdapterRemoval*.

The microbial community composition was evaluated with the bioinformatic tool MetaphlAn3 [43] at the phylum, class, order, family, genus, and species level. Alpha diversity was computed adopting the Shannon index implemented in the function ‘diversity’ of the *vegan* R-bioconductor package [44]. This package was also employed to calculate the Bray–Curtis beta distance (function ‘vegdist’) [44]. Beta values were transformed with the Classical multidimensional scaling function ‘cmdscale’ to perform the principal coordinate analysis (base package ‘stats’); the plot of 3D projections was then obtained using the ‘rgl’ utility.

### Statistical analysis

Growth performance data were analyzed through a one-way blocked ANOVA with the treatment as the experimental factor and the replicate pen as the experimental unit. Carcass and cut-up yields data were not statistically analyzed because they were recorded on a treatment basis. Count data of breast muscle myopathies, viz. WS, WB, and SM, were analyzed with Pearson’s Chi-squared test using the sampled animal as the experimental unit. Count data of breast muscle myopathies were also arranged in 2 by 2 contingency tables aligning the treatment levels (i.e., CON and ARG) and having binarily aggregated myopathy scores in columns (i.e., “presence” as a sum of score 1 and score 2 counts, while “absence” as score 0 counts). The incidence risk ratio was computed on these tables with the package *epiR* [45] of R [39]. If the incidence risk ratio was significant at 95% confidence interval, the risk of developing the myopathy was calculated as incidence risk ratio minus 1 and expressed in percentage [38].

A two-tailed Student’s *t*-test with the treatment as the experimental factor and the sampled bird as the experimental unit was carried out for the analysis of metabolomics data. Data deviating from normality in the Shapiro–Wilk test were subjected to Box-Cox transformation [46]. The abovementioned analyses were performed using R [39].

Concerning the caecal microbiota data, the relative frequency of abundance was computed and a two-sided Welch’s *t*-test was applied to highlight statistically significant differences between the treatments. Alpha diversity data were statistically analyzed with a two-tailed Student’s *t*-test. A representative boxplot for the genus level is shown in the result section.

*P*-values less than 0.05 were considered significant, while those ranging between 0.05 and 0.1 as tendencies.

## Results

### Growth performance

**Fig. 1:**
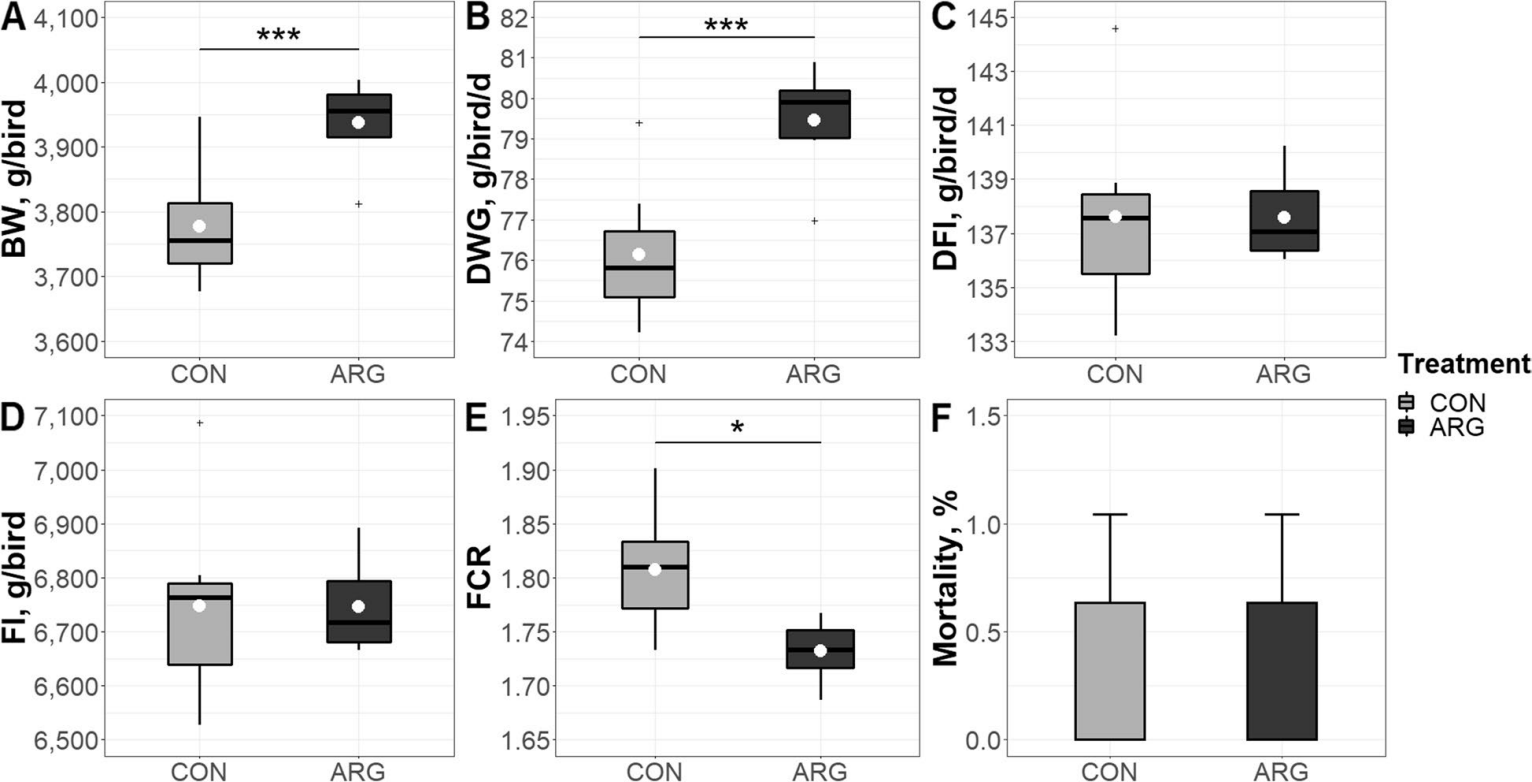
Final body weight (BW, **A**) and cumulative daily weight gain (DWG, **B**), daily feed intake (DFI, **C**), feed intake (FI, **D**), feed conversion ratio (FCR, **E**), and mortality (**F**) of CON and ARG birds in the entire trial (0–49 d). Means of 7 replicates/treatment are the white dots within the box plots or bar plots. ^*^P < 0.05; ^***^P < 0.001

Table 2 compares the growth performance of CON and ARG birds in the 4 feeding phases. At placement, chicks had an average weight of 40 g. At the end of the starter phase, ARG birds exhibited lower FCR than CON birds (1.345 and 1.303 for CON and ARG, respectively; *P* < 0.05), whereas other performance traits showed no differences between treatments. Likewise, ARG birds had the lowest FCR in the first grower phase (1.533 and 1.470 for CON and ARG, respectively; *P* < 0.05). No differences were detected in the second grower phase. At the end of the finisher phase, ARG birds showed greater BW than CON birds (3778 and 3937 g for CON and ARG, respectively; *P* < 0.001). Figure 1 illustrates the growth performance of CON and ARG birds in the entire feeding trial (0–49 d). While final BW and cumulative DWG were higher for ARG birds (3778 and 76.15 g, and 3937 and 79.46 g for CON and ARG, respectively; *P* < 0.001; Fig. 1A–B), their cumulative FCR was lower than that of CON birds (1.808 and 1.732 for CON and ARG, respectively; *P* < 0.05; Fig. 1E).

**Table 2:** Growth performance of CON and ARG birds at the end of each feeding phase

**Trait**	**Treatment**^a^		**SE**	***P*-value**
	**CON**	**ARG**		
Chick weight, g/bird	40.17	40.01	0.34	0.409
Starter (0–9 d)				
BW, g/bird	214.9	218.9	6.77	0.315
DWG^b^, g/d	19.42	19.88	0.74	0.293
DFI^b^, g/d	26.12	25.89	0.85	0.631
FI^b^, g/bird	235.1	233.0	7.62	0.631
FCR^b^	1.345	1.303	0.03	**0.044**
Mortality, %	0	0	0	/
Grower I (10–21 d)				
BW, g/bird	893.2	917.6	27.63	0.149
DWG^b^, g/d	56.52	58.22	1.94	0.152
DFI^b^, g/d	86.61	85.56	1.25	0.169
FI^b^, g/bird	1039	1027	15.05	0.169
FCR^b^	1.533	1.470	0.04	**0.018**
Mortality, %	0	0	0	/
Grower II (22–35 d)				
BW, g/bird	2262	2339	89.06	0.157
DWG^b^, g/d	97.79	101.5	4.75	0.190
DFI^b^, g/d	169.2	168.5	1.98	0.542
FI^b^, g/bird	2369	2359	27.65	0.542
FCR^b^	1.736	1.661	0.11	0.238
Mortality, %	0.32	0	0.59	0.356
Finisher (36–49 d)				
BW, g/bird	3778	3937	49.26	** < 0.001**
DWG^b^, g/d	108.0	114.0	7.22	0.172
DFI^b^, g/d	221.8	223.4	5.27	0.585
FI^b^, g/bird	3105	3127	73.72	0.585
FCR^b^	2.063	1.961	0.12	0.168
Mortality, %	0.32	0.64	0.59	0.356

^a^Mean of 7 replicates/treatment

^b^Corrected for mortality

*SE* standard error, *BW* body weight, *DWG* daily weight gain, *DFI* daily feed intake, *FI* feed intake, *FCR* feed conversion ratio

### Processing yields and breast muscle myopathies

**Fig. 2:**
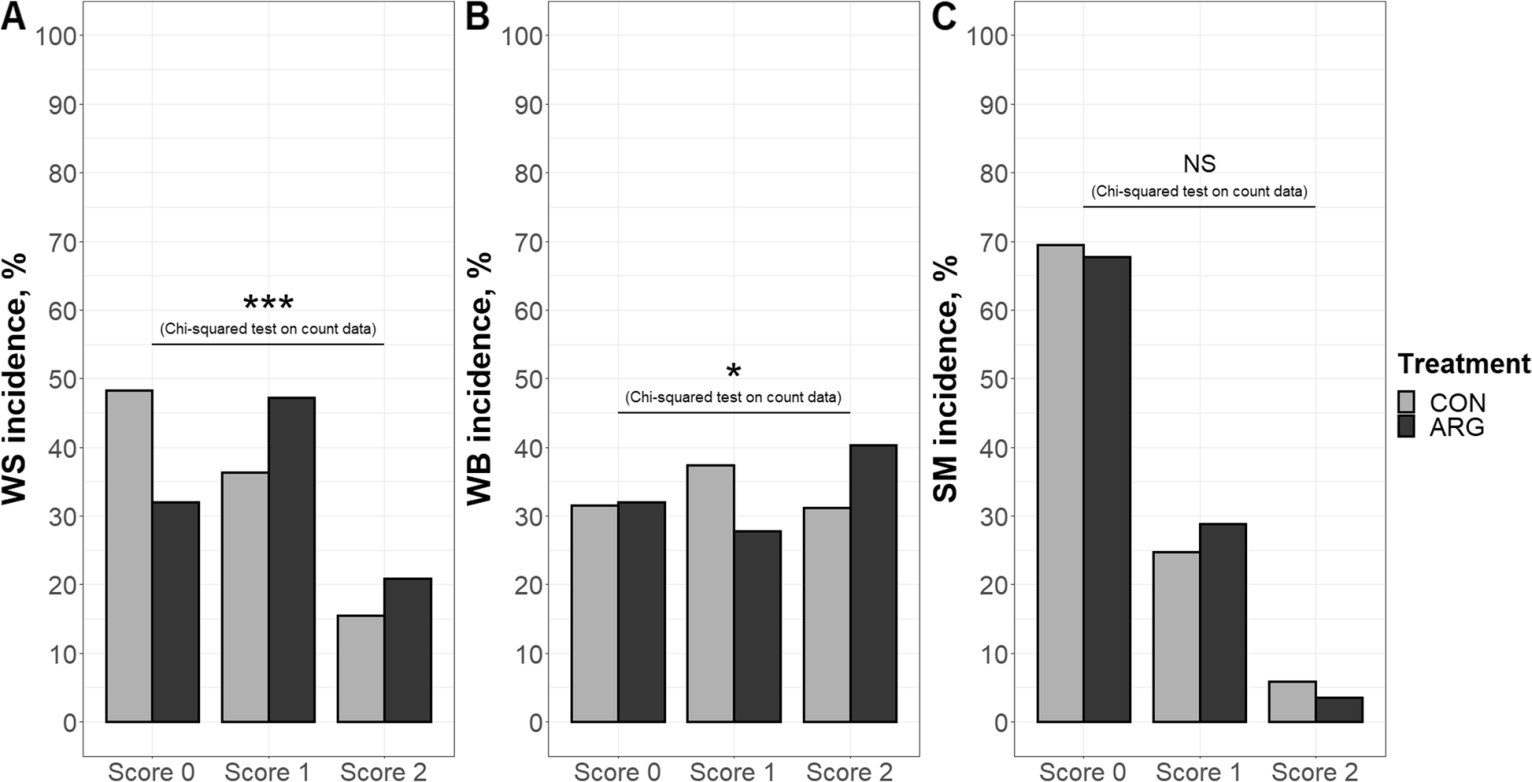
White striping (WS, **A**), woody breast (WB, **B**), and spaghetti meat (SM, **C**) incidence and severity in CON and ARG birds at D49. *n* = 292 and 288 breast fillets for CON and ARG, respectively. Score 0, normal; score 1, mild myopathy; score 2, severe myopathy. Count data were analyzed via Pearson’s Chi-squared test. ^*^P < 0.05; ^***^P < 0.001; NS, not significant

Carcass and breast yields – the latter calculated as percentage of the eviscerated carcass weight – of CON and ARG birds were similar (i.e., 73.5% and 33.4%, and 73.6% and 33.7%, for CON and ARG, respectively) and in line with the goals set by the breeding company [47]. Regarding breast muscle myopathies, the incidence of WS and WB was related to the factor treatment (*P* < 0.001 and *P* < 0.05 for WS and WB, respectively), with ARG birds exhibiting a higher incidence of mild WS and severe WS and WB than CON birds (Fig. 2A–B). However, the incidence risk ratio analysis revealed that arginine supplementation had a significant effect only on WS onset: ARG birds were 1.32 (95% confidence interval of 1.15 to 1.51) times more likely to develop WS than CON birds; that is, feeding the arginine-supplemented diet significantly increased by 32% the relative risk of developing WS. On the other hand, SM did not show a significant association with the factor treatment (Fig. 2C).

### Plasma, liver, and caecal content metabolic profiles

A total of 60, 71, and 78 metabolites were identified in plasma, liver, and caecal content samples, respectively. Table 3 presents metabolites showing different concentrations between CON and ARG birds. In the plasma, ARG birds had a significantly lower concentration of 2-oxoglutarate, glutamine, and methanol, while fumarate and mannose showed a comparable but not significant trend. In addition, the concentrations of arginine, betaine, and histidine were significantly greater for ARG birds, with acetate and creatine exhibiting a similar tendency toward significance. In the liver, 7 metabolites showed a different concentration between treatments. While glutathione displayed a decreasing trend in ARG birds, aspartate, creatine, leucine, phenylalanine, and threonine varied in the opposite way. Furthermore, the concentration of methionine sulfoxide was significantly higher in ARG birds. In the caeca, ARG birds showed a significantly lower concentration of leucine and an almost significant increase in thymine than CON birds.

**Table 3:** Metabolites showing different concentrations (mmol/L) in the plasma, liver, and caecal content of CON and ARG birds at D49

**Metabolite**	**Treatment**^a^		**SE**	***P*-value**	**Variation**^**b**^
	**CON**	**ARG**			
Plasma					
2-Oxoglutarate	9.64E-02	7.67E-02	1.61E-02	0.004	↓
Acetate	4.37E-02	5.94E-02	2.23E-02	0.079	↑
Arginine	3.25E-01	4.09E-01	1.38E-02	0.018	↑
Betaine	6.06E-01	6.90E-01	8.62E-02	0.023	↑
Creatine	7.67E-02	9.58E-02	8.96E-02	0.060	↑
Fumarate	1.39E-02	1.22E-02	2.83E-02	0.057	↓
Glutamine	1.46E + 00	1.32E + 00	2.22E-03	0.043	↓
Histidine	9.97E-02	1.17E-01	1.77E-01	0.009	↑
Mannose	4.30E-02	3.95E-02	1.56E-02	0.066	↓
Methanol	5.35E-02	4.19E-02	4.67E-03	0.026	↓
Liver					
Aspartate	5.02E-03	5.81E-03	1.14E-03	0.078	↑
Creatine	3.60E-04	5.40E-04	2.44E-04	0.056	↑
Glutathione	1.50E-04	6.00E-05	1.09E-04	0.064	↓
Leucine	1.58E-03	2.00E-03	5.82E-04	0.066	↑
Methionine sulfoxide	2.00E-05	3.00E-05	7.05E-06	0.034	↑
Phenylalanine	7.90E-04	1.02E-03	3.03E-04	0.062	↑
Threonine	1.77E-03	2.12E-03	5.41E-04	0.094	↑
Caecal content					
Leucine	2.47E-03	1.79E-03	7.53E-04	0.017	↓
Thymine	4.80E-04	6.50E-04	2.29E-04	0.051	↑

^a^Mean of 14 birds/treatment

^b^Ratio of CON mean over ARG mean: ↑, ratio < 1; ↓, ratio > 1

*SE* standard error

### Caecal microbiota

**Fig. 3:**
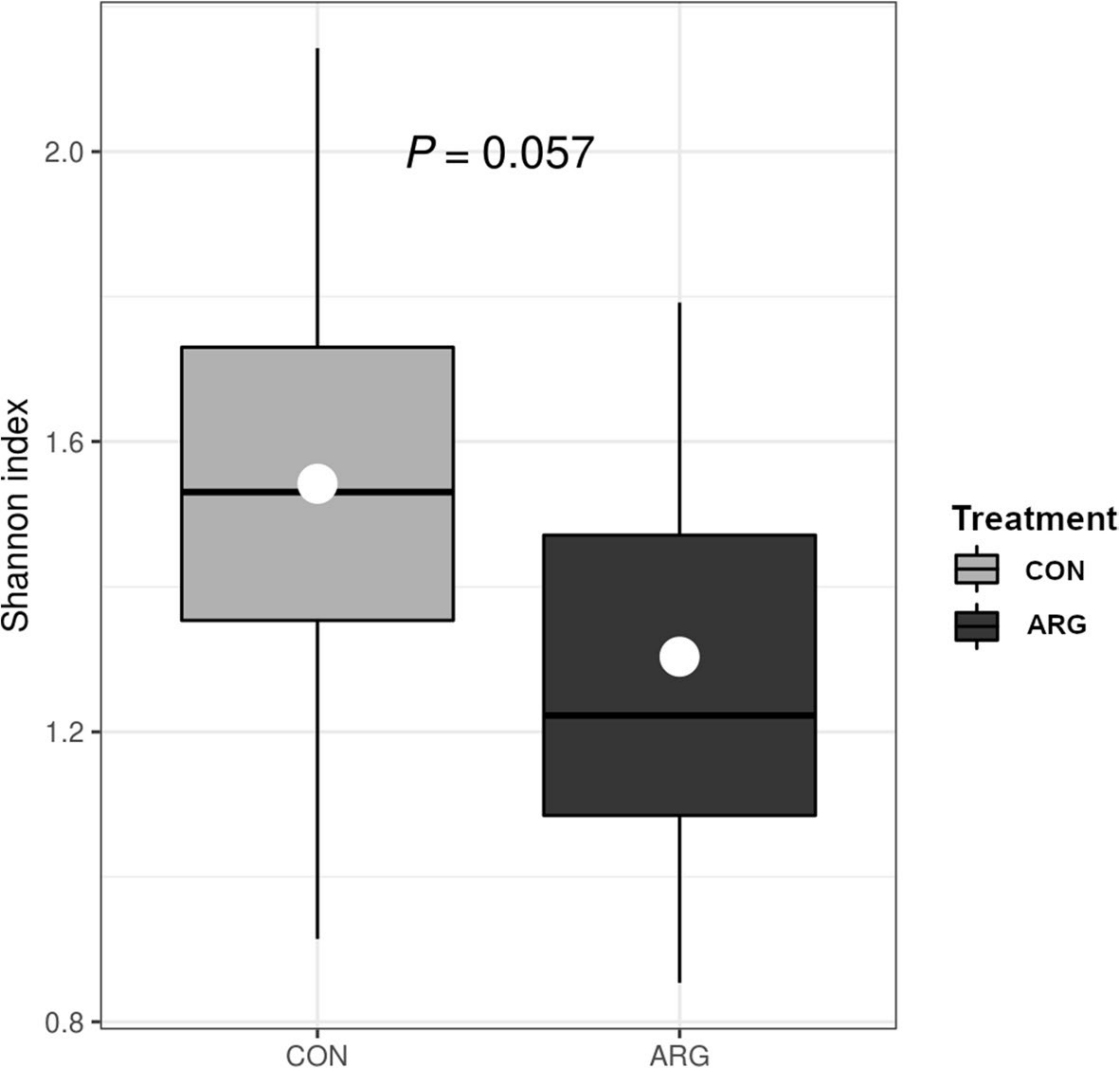
Shannon index in the caecal content of CON and ARG birds at D49. Means of 14 birds/treatment are the white dots within the box plots

Alpha diversity of ARG caecal content samples tended to be lower than that of CON samples at almost all taxonomic levels, except for the species (see Additional file 1). At the genus level, ARG samples had an average alpha diversity of 1.3 and CON samples of 1.5 (*P* = 0.06) as illustrated in Fig. 3. The beta diversity analysis did not cluster the samples according to treatments (see Additional file 2). Table 4 shows bacteria that were differently abundant in caecal content samples of CON and ARG birds at D49. At the phylum level, the relative abundance of Proteobacteria was significantly lower in ARG than CON birds (*P* < 0.05), while Firmicutes had a similar trend toward significance. Bacteroidetes, in contrast, were overrepresented in ARG compared to CON birds (*P* < 0.05). At the class level, Bacteroidia were significantly more abundant in ARG than CON birds, as were Coriobacteriia – a class of the phylum Actinomycetota – whereas the relative abundance of Gammaproteobacteria, unclassified Firmicutes, and Clostridia changed in the opposite way (*P* < 0.05). The differences in relative abundance detected at the order level reflected those at the class level: Bacteroidales and Eggerthellales (the latter belonging to Coriobacteriia class) were significantly more abundant, while Enterobacterales (members of Gammaproteobacteria class), unclassified Firmicutes, and Clostridiales were less abundant in ARG than CON birds (*P* < 0.05). Moving to bacterial families, the relative abundance of Eggerthellaceae was higher in ARG than CON birds (*P* < 0.05). On the other hand, the relative abundance of Enterobacteriaceae and unclassified Firmicutes was significantly lower in ARG than CON birds (*P* < 0.05). With respect to bacterial genera, ARG birds showed a greater abundance of *Gordonibacter* (*P* < 0.05) and a lower abundance of *Escherichia*, unclassified Firmicutes, and *Flavonifractor* (0.06 ≤ *P* ≤ 0.02). Lastly, the bacterial species whose relative abundance differed between treatments were *Gordonibacter pamelaeae* and *Lactobacillus salivarius* (significantly more abundant in ARG birds; *P* < 0.05), along with *Escherichia coli*, *Firmicutes bacterium* CAG 94, and *Lachnoclostridium* An131 (less abundant in ARG birds with a *P*-value ranging between 0.09 and 0.03).

**Table 4:** Bacteria showing different relative abundance (%) in the caecal content of CON and ARG birds at D49

**Bacteria**	**Treatment**^a^		***P*-value**	**Variation**^**b**^
	**CON**	**ARG**		
Phylum				
Proteobacteria	1.70	0.14	0.018	↓
Bacteroidetes	72.3	80.9	0.041	↑
Firmicutes	19.0	14.6	0.087	↓
Class				
Coriobacteriia	0.19	0.42	0.010	↑
Gammaproteobacteria	1.70	0.14	0.018	↓
Firmicutes (unclassified)	4.80	2.58	0.041	↓
Bacteroidia	72.3	80.9	0.041	↑
Clostridia	13.5	9.35	0.043	↓
Order				
Eggerthellales	0.19	0.42	0.010	↑
Enterobacterales	1.70	0.14	0.018	↓
Firmicutes (unclassified)	4.80	2.58	0.041	↓
Bacteroidales	72.3	80.9	0.041	↑
Clostridiales	13.5	9.35	0.043	↓
Family				
Eggerthellaceae	0.19	0.42	0.010	↑
Enterobacteriaceae	1.70	0.14	0.018	↓
Firmicutes (unclassified)	4.80	2.58	0.041	↓
Genus				
* Gordonibacter*	0.19	0.42	0.010	↑
* Escherichia*	1.70	0.14	0.018	↓
* Firmicutes* (unclassified)	4.80	2.58	0.041	↓
* Flavonifractor*	1.57	0.81	0.059	↓
Species				
* Gordonibacter pamelaeae*	0.19	0.42	0.010	↑
* Escherichia coli*	1.70	0.14	0.018	↓
* Lactobacillus salivarius*	0.01	0.06	0.030	↑
* Firmicutes bacterium* CAG 94	4.80	2.58	0.041	↓
* Lachnoclostridium* An131	0.33	0.13	0.091	↓

^a^Mean of 14 birds/treatment

^b^Ratio of CON mean over ARG mean: ↑, ratio < 1; ↓, ratio > 1

## Discussion

Besides serving as a building block for protein synthesis, arginine is the precursor of compounds that exert a myriad of biological effects and represents the secretagogue for fundamental hormones [3]. These properties place arginine at the center of vital physiological processes and emphasize the importance of satisfying its requirements in broiler nutrition. The results obtained from this study confirm that feeding arginine above the recommended specifications improves the growth performance of broilers. The significantly lower FCR showed by birds receiving arginine supplementation – particularly up to D21 (−4.1%) and in the overall trial (−4.2%) – is in line with our earlier findings [48], while the substantial increase in their cumulative DWG (+ 4.4%) and final BW (+ 4.2%) corroborates the data of other research groups [49–52]. Nevertheless, Kidd et al. [53] found an inconsistent response of BW gain to dietary arginine levels exceeding the amounts recommended by the NRC, while some investigators did not detect any improvement in BW when supplementing broiler diets with extra arginine [54, 55]. Therefore, additional research may be necessary to validate the positive effects of arginine supplementation on BW gain of broiler chickens.

The macroscopic analysis of breast fillets showed that supplemental arginine increased by 32% the risk of WS onset. This outcome is contrary to previous studies reporting no significant effects of dietary arginine supplementation on WS occurrence, or even a mitigation of this breast muscle myopathy [48, 56–58]. However, in contrast to the substantial improvement in growth rate and BW observed here, it should be noted that arginine supplementation tested in the studies just cited did not exert a positive effect on BW gain. Previous research has established that breast muscle abnormalities of fast-growing, high meat-yielding broilers are deeply related to their extraordinary growth potential: the higher the growth performance, the greater the risk of myopathy onset [59–61]. It is therefore very likely that the significant arginine-mediated growth promotion exacerbated WS rather than the supplemental dietary arginine per se.

In addition to the evaluation of the effects on growth performance, the second aim of the current study was to investigate the impacts of arginine supplementation on metabolism and intestinal microbiota of broilers. Plasma of ARG birds showed a significantly higher concentration of arginine than that of CON birds. This result supports those formerly reported by our group [48], Kidd et al. [53], and researchers working with piglets [62] and rats [63]. Therefore, it is conceivable that feeding broilers arginine above the typical recommended levels is an effective way to increase dietary arginine bioavailability. This can be of paramount importance for animals incapable of synthesizing arginine de novo [64]. In addition, extra dietary arginine has been shown to stimulate the secretion of GH, IGF-1, and insulin in broilers [50, 52] and piglets [62]. In ARG birds, high plasma concentrations of arginine may have indirectly boosted the anabolic pathways through those potent hormones [14–17].

Arginine intake and availability have also been demonstrated to influence creatine levels in different parts of the chicken's body [65–67]. Interestingly, our metabolomics analyses revealed greater concentrations of circulating and hepatic creatine in ARG birds. Creatine is mainly produced by the liver and is subsequently delivered to target tissues through the bloodstream [19, 68]. In light of this, it can be supposed that the skeletal muscle of ARG birds had a higher creatine content than that of CON birds, as previously proved by Chamruspollert et al. [67]. Extensive research has shown that supplementing creatine – or its precursor, guanidinoacetate – considerably improves growth performance and breast meat yield of broilers [19, 69, 70]. Consequently, increased creatine availability may have supported the growth and lean tissue accretion for ARG birds. However, the evaluation of lean tissue yield was beyond the scope of the present study, hence our hypothesis is to be confirmed by experimental data.

Besides arginine and creatine, ARG birds exhibited higher plasma concentration of histidine – which confirms our previous study [48] – and betaine than CON birds. Plasma histidine level has been shown to be positively correlated with the *Pectoralis major* weight [71], while feeding broilers on a histidine-deficient diet resulted in impaired growth, reduced breast meat yield, and complete carnosine depletion and a significant decrease in anserine in pectoral muscle [72]. On the other hand, supplementation of dietary histidine increased breast muscle content of histidine-containing dipeptides, viz. carnosine and anserine, thereby improving the quality and antioxidant defenses of chicken meat [72, 73]. Future research is warranted to elucidate the causes and effects of the increased plasma histidine level observed in arginine-supplemented broilers. As for betaine, several lines of evidence suggest that it enhances the health, performance, carcass composition, and meat quality of poultry [74]. Thanks to its osmoregulatory function, betaine can also mitigate the detrimental effects of heat stress [75]. Furthermore, acting as a methyl group donor, betaine contributes to and promotes the biosynthesis of creatine in the liver of broilers [76]. The higher availability of betaine is therefore another plausible reason for the greater hepatic creatine content creatine and better performance of ARG birds than their control counterparts.

Not only the plasma metabolic profile, but also the hepatic one appeared to be affected by the arginine supplementation tested here. Along with creatine, the liver of ARG birds was enriched in leucine, methionine sulfoxide, phenylalanine, and threonine, which are all indispensable amino acids for chickens [77]. It is intriguing to link the increased hepatic levels of these essential amino acids to potentially better intestinal digestion and absorption of dietary protein and purified amino acids, such as crystalline methionine and threonine included in the basal diet used in this study. Indeed, arginine supplementation has been shown to improve intestinal health, integrity, and function [21, 23, 26, 27] and to increase the jejunal villus height to crypt depth ratio in broilers [78] and jejunal and ileal villus height in intra-uterine growth retarded piglets [79]. The villus height to crypt depth ratio is commonly used to assess chicken gut health [80], while villi extend nutrient absorption area per se [81]. These findings reported in the literature led us to suppose that improved intestinal conditions and desirable changes in gut morphology may have been behind an elevated efficiency of amino acid uptake in ARG birds. Since nutrient absorption primarily occurs in the jejunum and amino acids are not assimilated through the large intestine epithelium [82, 83], the fact that CON birds showed increased – probably unabsorbed – level of leucine in the caecal content suggests that the small intestine uptake of leucine might have been higher for ARG birds, further supporting our hypothesis. Additional research (e.g., digestibility studies) focused on this topic is therefore suggested.

Furthermore, ARG birds had less hepatic glutathione than CON birds. Glutathione, a tripeptide composed of glutamate, cysteine, and glycine, plays a pivotal role in the antioxidant defense system, metabolism of nutrients, and regulation of cellular activities. As previously seen for creatine, the liver is the most important producer and provider of glutathione [13]. Being the precursor of glutamate, arginine influences the biosynthesis and levels of glutathione [4]. Enkvetchakul et al. [84] described an age- and body weight-dependent increase in hepatic and blood glutathione for broiler chickens. Given that CON and ARG birds were equal in age at sampling (D49), we would have expected a greater glutathione level in the liver of heavier broilers, namely ARG birds. However, the outcome opposite to expectations raises the very interesting question of why arginine supplementation may have produced a reduction in hepatic glutathione for ARG birds. It is difficult to answer this query, especially in view of the dietary arginine-supported increase in glutathione peroxidase activity found in broiler breeder hens and their offspring [85]. Another unanswered question is: can the increased concentration of methionine sulfoxide, the major product of methionine oxidation [86], represent an indicator for hepatic oxidative stress in ARG birds? In the liver, methionine can be converted into cysteine, which is the rate-limiting amino acid for glutathione synthesis [13, 87]. Interestingly, feeding aflatoxin-challenged broilers a diet supplemented with methionine caused an unforeseen attenuation in hepatic glutathione [87]. Conversely, other investigators found that methionine supplementation increased glutathione in the liver and intestinal mucosa of broilers [88, 89]. Further studies could shed light on the modifications in glutathione concentrations induced by arginine and methionine supplementation in broiler diets.

Turning now to the results of the caecal microbiota analysis, it was observed that arginine supplementation reduced alpha diversity. This finding is contrary to that of Singh et al. [28] who measured an increase in Shannon index for colonic specimens of mice given high dietary arginine compared to their low-dose and control counterparts. It is reasonable to attribute this discrepancy to the different animal species used (i.e., chicken vs. mouse) and the intestinal section the digesta was collected from (i.e., caecum vs. colon). Caecal samples of ARG birds also showed a decrease in the relative abundance of Firmicutes (e.g., Clostridia) and an increase in the relative abundance of Bacteroidetes (e.g., Bacteroidia). We have recently reported a comparable reduction in caecal alpha diversity and a similar change in the abundance of Firmicutes and Bacteroidetes for high-performing broilers treated with a feed-grade muramidase [38]. Although it is important to take into consideration the differences between the present study and our previous one [38], these findings somewhat contradict the widely known favorable association between microbial diversity or relative abundance of Firmicutes (especially useful Clostridia) and broiler health and performance [90–92]. Remarkably, Singh et al. [28] also found increased prevalence of Bacteroidetes in the colon of mice fed on a high-arginine diet. Thus, these topics are worth investigating further in chickens. Proteobacteria were also affected by the arginine supplementation utilized in the present study. Specifically, the relative abundance of *E. coli* was lower in ARG than CON birds. This is consistent with the results obtained in a murine model of *E. coli* infection, wherein arginine supplementation was tested as a potential therapy [93]. The authors of the latter paper suggested that arginine supplementation attenuated *E. coli* infection by means of a positive regulation of the intestinal innate immunity. Moreover, Liu et al. [21] proved that arginine supplementation alleviated gut mucosal injury in weaned piglets challenged with lipopolysaccharide from *E. coli, *ascribing this beneficial outcome to a possible immunomodulatory effect of arginine. Likewise, Guo’s lab demonstrated that arginine supplementation mitigated intestinal damage in *Clostridium perfringens*-infected broilers by enhancing mucosal barrier and immune function, increasing nitric oxide production, and restoring a normal microbiota [26, 27]. So, we believe that it is worth delving into the potentially desirable effects of arginine supplementation on the intestinal immune function of broilers.

Despite the reduction in Firmicutes, ARG birds had a higher relative abundance of *Lactobacillus salivarius*, which has extensively been studied for its probiotic effects and has frequently been used as a feed additive to improve the health and performance of livestock and poultry [94, 95]. Taken together, the microbiota results of our investigation indicate that arginine supplementation may have induced advantageous changes in the intestinal ecosystem of broilers, with possible beneficial implications for gut health, systemic health, and growth performance. However, as recommended by Singh et al. [28], further work needs to be done to clarify how arginine supplementation influences the gut microbiota and its relationship with the host.

## Conclusions

The present study confirms that formulating diets with high levels of arginine (i.e., total arginine to total lysine ratio of 1.20 instead of 1.06–1.08 recommended by the breeding company) is beneficial for the productive performance of fast-growing broilers. However, due to inconsistent data found in the literature, the positive effect on the final body weight may entail further proofs. The observed improvement in growth performance is likely to be related to increased availability of arginine, betaine, histidine, and creatine in the plasma and liver, as well as to the ability of dietary arginine to potentially ameliorate intestinal conditions and microbiota. The latter promising property, however, raises intriguing questions about the mechanism by which supplemental dietary arginine modulates the intestinal ecosystem and host-microbiota interactions in broilers (i.e., direct or indirect effects?). Overall, this study offers valuable insights into the metabolic and microbiota changes occurring in broilers fed diets with arginine concentrations beyond the recommended levels, which can pave the way for more specific investigations.

## Supplementary Information

**Additional file 1.** Alpha (Shannon) and beta diversity values of caecal content samples of CON and ARG birds at D49.

**Additional file 2.** Relative abundances of bacteria and plots of alpha (Shannon) and beta diversity of caecal content samples of CON and ARG birds at D49.

## Data Availability

All data generated and analyzed in this study have been included in the present article and its additional files. The metagenomes are available in the SRA repository [BioProject PRJNA884508, https://dataview.ncbi.nlm.nih.gov/object/PRJNA884508?reviewer=t7u6lk5fa366vfuc3qam3p701e].

## Abbreviations

ARG: Arginine group fed the basal diet supplemented with *L*-arginine
BW: Body weight
CON: Control group fed the basal diet
DFI: Daily feed intake
DWG: Daily weight gain
FCR: Feed conversion ratio
FI: Feed intake
GH: Growth hormone
IGF-1: Insulin-like growth factor-1
mTOR: Mechanistic target of rapamycin
SE: Standard error
SM: Spaghetti meat
WB: Woody breast
WS: White striping
