# Supplementary material for: Feeding broiler chickens with arginine above recommended levels: effects on growth performance, metabolism, and intestinal microbiota
Source: J Anim Sci Biotechnol. 2023 Mar 3;14:33. doi: 10.1186/s40104-023-00839-y (PMC9983211; doi:10.1186/s40104-023-00839-y)

EVALUATION OF TAXA COMPOSITION

All values refer to relative abundance expressed as percentage

Phylum level

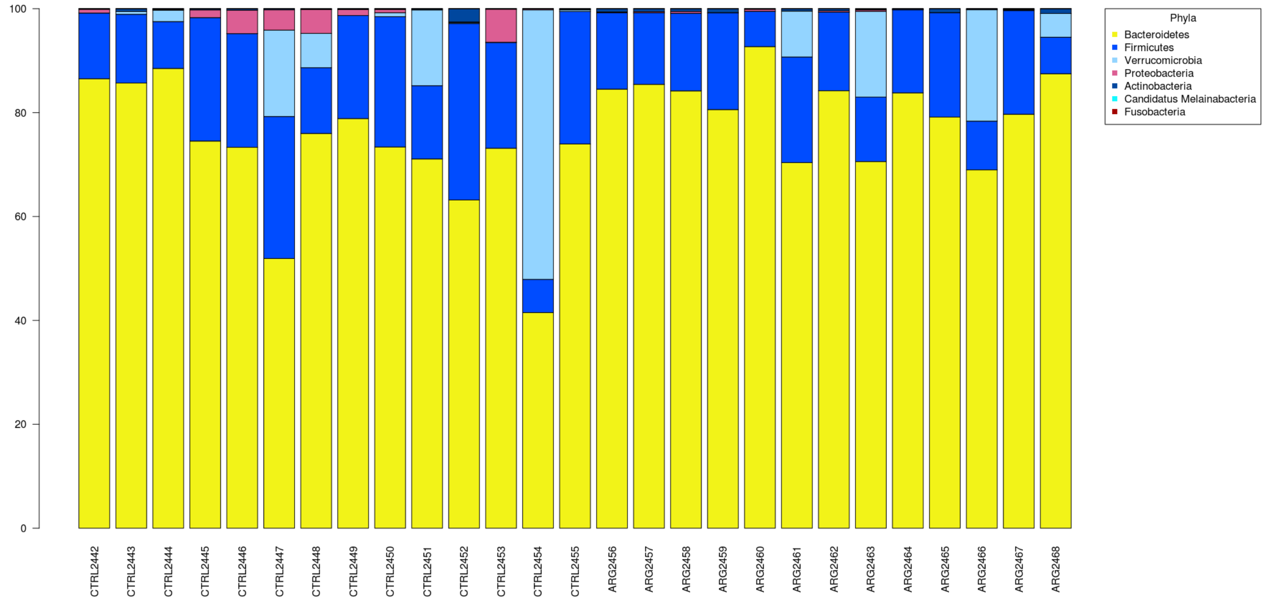

PHYLUM	p.value	Media Trattati ARG	Media CTRL
**Proteobacteria**	**0.01828863**	**0.14057**	**1.70134**
**Bacteroidetes**	**0.04144114**	**80.89851**	**72.25759**
Firmicutes	0.08673245	14.54999	18.97921
Candidatus Melainabacteria	0.3546591	0.00711	0.00032
Verrucomicrobia	0.53040785	3.96129	6.69495
Actinobacteria	0.68913423	0.44188	0.36556
Fusobacteria	0.76420152	0.00066	0.00104

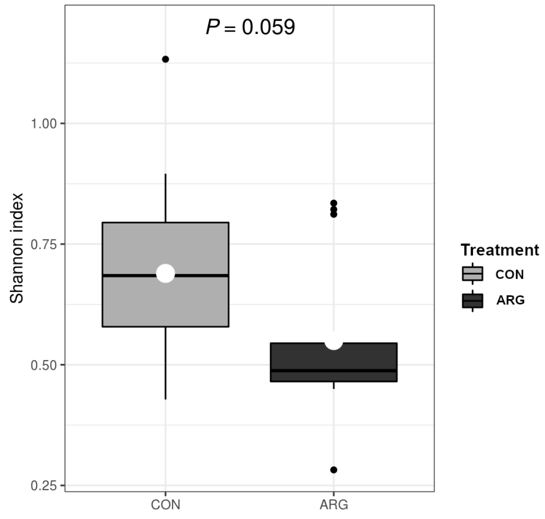

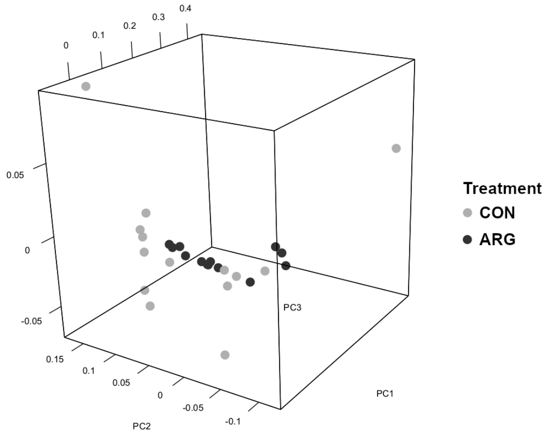

Class-level

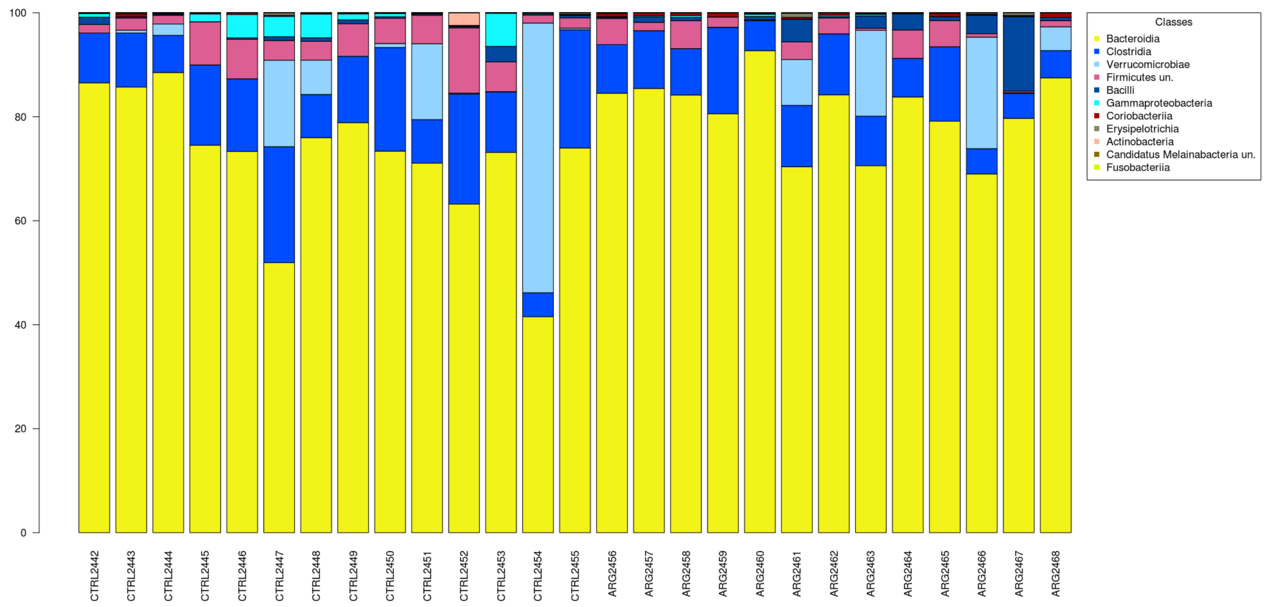

CLASS	p.value	Media Trattati ARG	Media CTRL
**Coriobacteriia**	**0.009920477**	**0.42424**	**0.18823**
**Gammaproteobacteria**	**0.01828863**	**0.14057**	**1.70134**
**Firmicutes un.**	**0.041153297**	**2.58153**	**4.79912**
**Bacteroidia**	**0.041441136**	**80.89851**	**72.25759**
**Clostridia**	**0.042522647**	**9.35020**	**13.45959**
Bacilli	0.116991405	2.45785	0.63593
Erysipelotrichia	0.33592567	0.16040	0.08456
Candidatus Melainabacterian un.	0.354659102	0.00711	0.00032
Actinobacteria	0.376435028	0.01764	0.17733
Verrucomicrobiae	0.530407851	3.96129	6.69495
Fusobacteriia	0.764201525	0.00066	0.00104

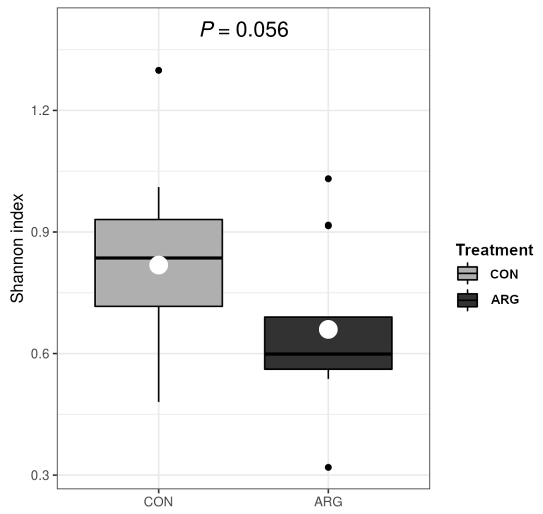

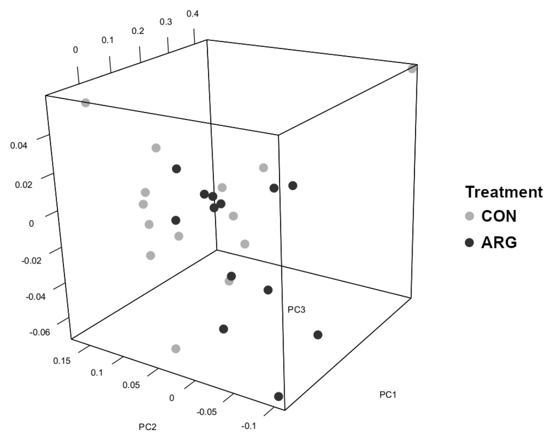

Order-level

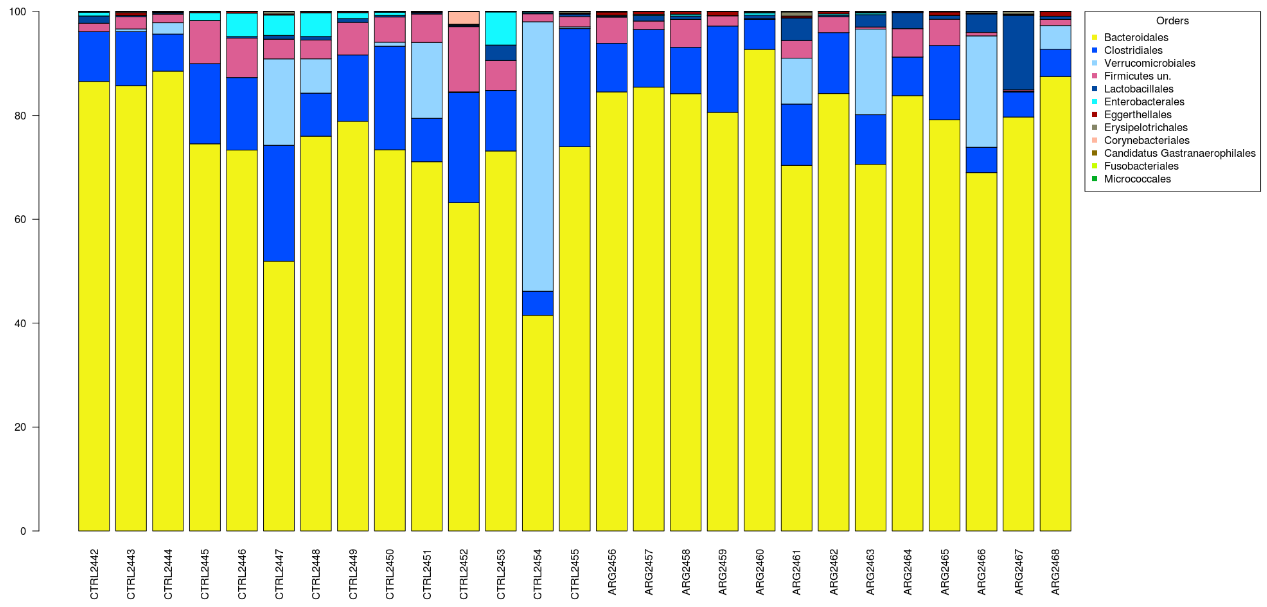

ORDER	p.value	Media Trattati ARG	Media CTRL
**Eggerthellales**	**0.009920477**	**0.42424**	**0.18823**
**Enterobacterales**	**0.01828863**	**0.14057**	**1.70134**
**Firmicutes un.**	**0.041153297**	**2.58153**	**4.79912**
**Bacteroidales**	**0.041441136**	**80.89851**	**72.25759**
**Clostridiales**	**0.042522647**	**9.35020**	**13.45959**
Lactobacillales	0.116991405	2.45785	0.63593
Micrococcales	0.335561278	0.00000	0.00043
Erysipelotrichales	0.33592567	0.16040	0.08456
Candidatus Gastranaerophilales	0.354659102	0.00711	0.00032
Corynebacteriales	0.376542311	0.01764	0.17690
Verrucomicrobiales	0.530407851	3.96129	6.69495
Fusobacteriales	0.764201525	0.00066	0.00104

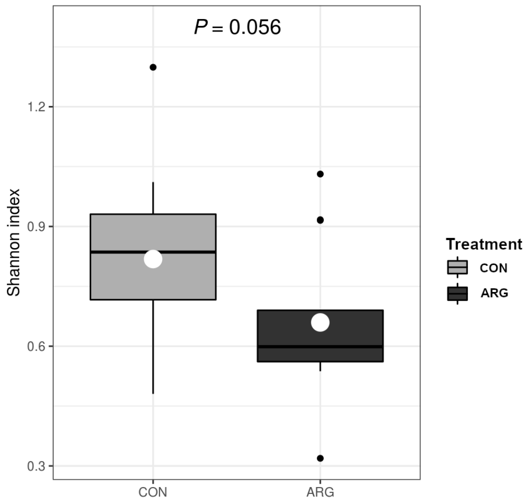

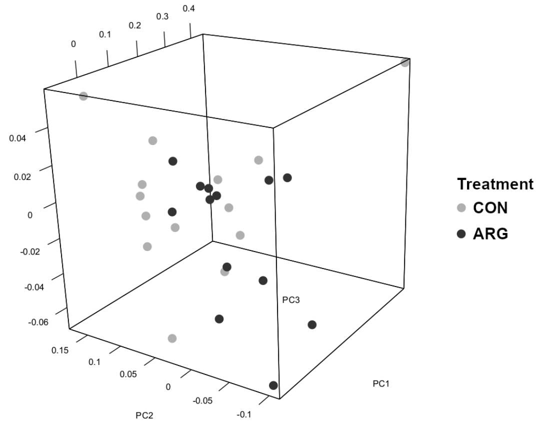

Family-level

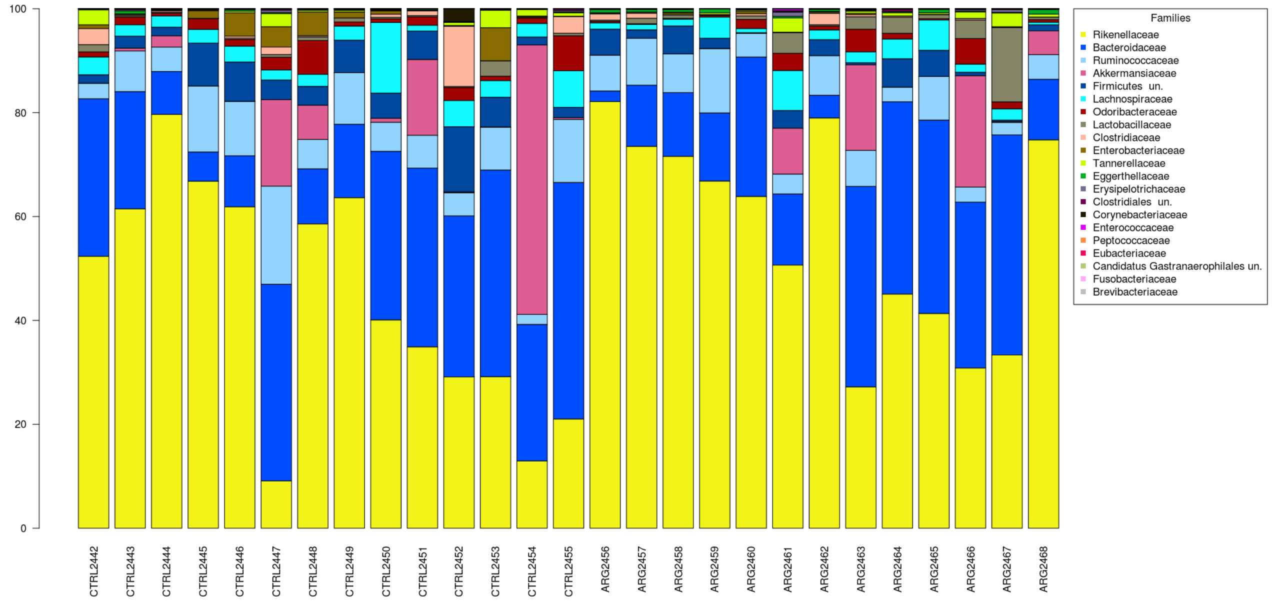

FAMILY	p.value	Media Trattati ARG	Media CTRL
**Eggerthellaceae**	**0.009920477**	**0.42424**	**0.18823**
**Enterobacteriaceae**	**0.01828863**	**0.14057**	**1.70134**
**Firmicutes un.**	**0.041153297**	**2.58153**	**4.79912**
Lactobacillaceae	0.117967044	2.41936	0.61079
Rikenellaceae	0.129209569	56.93586	44.34486
Peptococcaceae	0.181439715	0.01195	0.00038
Clostridiaceae	0.208460046	0.45979	1.57036
Ruminococcaceae	0.221471646	6.15262	7.98823
Lachnospiraceae	0.268211395	2.61782	3.79631
Brevibacteriaceae	0.335561278	0.00000	0.00043
Erysipelotrichaceae	0.33592567	0.16040	0.08456
Candidatus Gastranaerophilales un.	0.354659102	0.00711	0.00032
Corynebacteriaceae	0.376542311	0.01764	0.17690
Odoribacteraceae	0.381672675	1.47356	2.10377
Akkermansiaceae	0.530407851	3.96129	6.69495
Bacteroidaceae	0.557471897	21.75909	24.89844
Tannerellaceae	0.654598391	0.73000	0.91053
Enterococcaceae	0.679850152	0.03850	0.02514
Eubacteriaceae	0.729765742	0.00512	0.00304
Fusobacteriaceae	0.764201525	0.00066	0.00104
Clostridiales un.	0.970401238	0.10292	0.10129

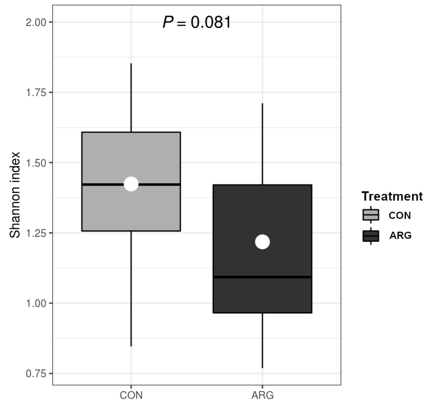

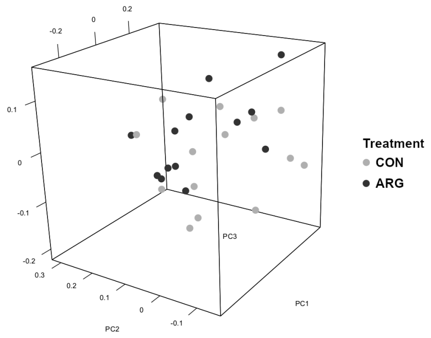

Genus-level

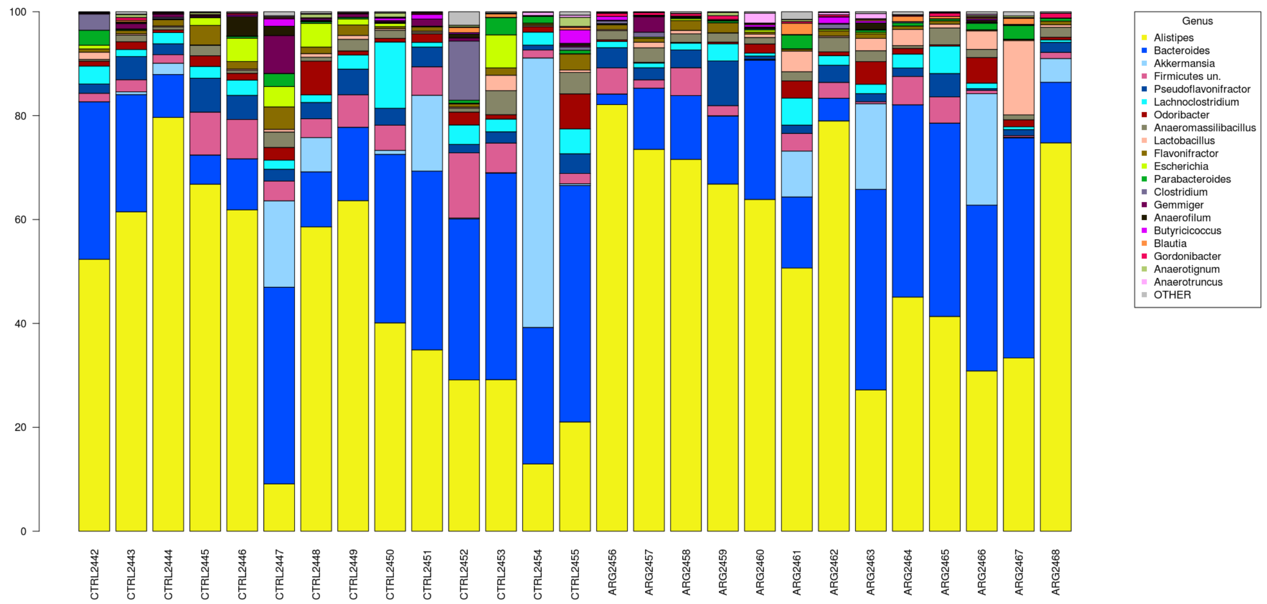

GENUS	p.value	Media Trattati ARG	Media CTRL
**Gordonibacter**	**0.009897337**	**0.42401**	**0.18823**
**Escherichia**	**0.01828863**	**0.14057**	**1.70134**
**Firmicutes un.**	**0.041153297**	**2.58153**	**4.79912**
Flavonifractor	0.05907798	0.80754	1.56574
Lactobacillus	0.117967044	2.41936	0.61079
Anaerotignum	0.127021007	0.13849	0.35911
Alistipes	0.129209569	56.93586	44.34486
Lachnoclostridium	0.194235344	2.01710	3.23248
Blautia	0.207605718	0.45106	0.19323
Anaerofilum	0.260537471	0.18029	0.52635
Clostridium	0.285339335	0.20486	1.12305
Gemmiger	0.286004706	0.29739	0.90399
Anaerotruncus	0.312035029	0.30298	0.13257
Odoribacter	0.381672675	1.47356	2.10377
Butyricicoccus	0.398800126	0.25493	0.44731
Pseudoflavonifractor	0.447193524	2.68035	3.24791
Akkermansia	0.530407851	3.96129	6.69495
Anaeromassilibacillus	0.537074711	1.85275	1.57490
Bacteroides	0.557471897	21.75909	24.89844
Parabacteroides	0.654598391	0.73000	0.91053
OTHER	0.794299158	0.38703	0.44134
Clostridiales un.	0.9577377	0.10292	0.10058
Erysipelatoclostridium	0.33592567	0.16040	0.08456
Corynebacterium	0.376542311	0.01764	0.17690

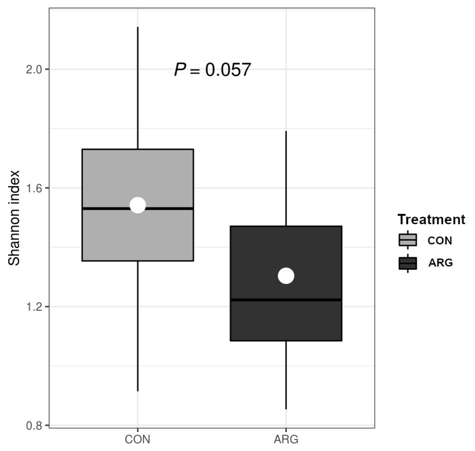

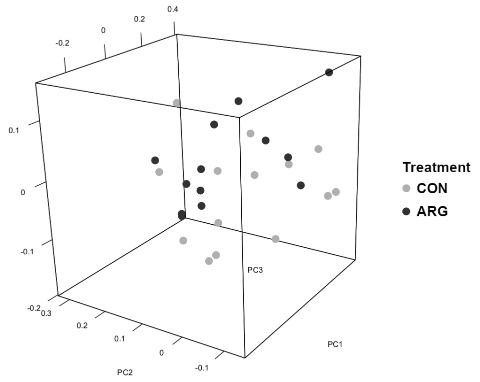

Specie-level

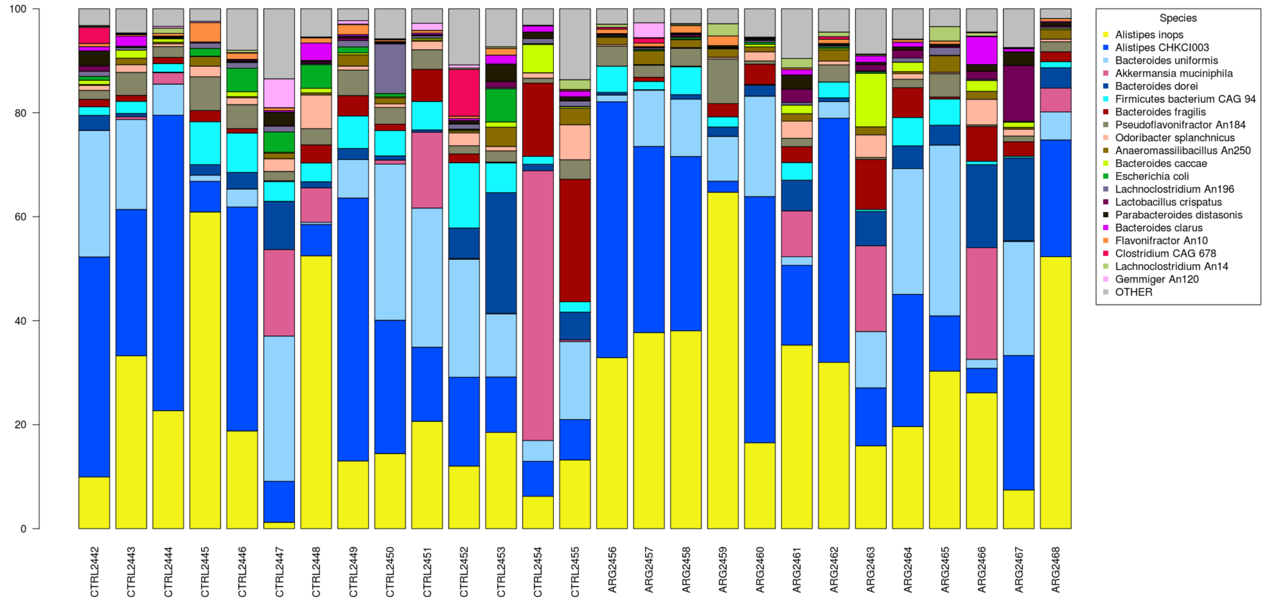

SPECIE	p.value	Media Trattati ARG	Media CTRL
**Gordonibacter pamelaeae**	**0.009897337**	**0.42401**	**0.18823**
**Escherichia coli**	**0.01828863**	**0.14057**	**1.70134**
**Lactobacillus salivarius**	**0.030119257**	**0.05873**	**0.00648**
**Firmicutes bacterium CAG 94**	**0.041153297**	**2.58153**	**4.79912**
Lachnoclostridium An131	0.090596564	0.13060	0.33314
Lachnoclostridium An118	0.104755833	0.24116	0.35846
Alistipes inops	0.113499734	31.45234	21.24149
Anaerotignum lactatifermentans	0.127021007	0.13849	0.35911
Lachnoclostridium An196	0.135969267	0.37242	1.39307
Lachnoclostridium An76	0.139583078	0.11211	0.28749
Lachnoclostridium An138	0.165207044	0.31065	0.52185
Lachnoclostridium An14	0.166764076	0.75170	0.32256
Lactobacillus crispatus	0.168770384	1.47455	0.29233
Flavonifractor An306	0.178086171	0.16455	0.58944
Blautia An249	0.207605718	0.45106	0.19323
Anaeromassilibacillus An200	0.208930728	0.22015	0.39433
Anaerofilum An201	0.260537471	0.18029	0.52635
Anaeromassilibacillus An250	0.270707072	1.58798	1.15526

Clostridium CAG 678	0.282625604	0.14466	0.89118
OTHER	0.296046587	4.82195	6.17051
Anaerotruncus colihominis	0.312035029	0.30298	0.13257
Flavonifractor An10	0.328924819	0.61370	0.90871
Odoribacter splanchnicus	0.381672675	1.47356	2.10377
Gemmiger An120	0.382074609	0.25498	0.65380
Butyricicoccus pullicaecorum	0.398800126	0.25493	0.44731
Pseudoflavonifractor An184	0.433437544	2.58300	3.18527
Bacteroides fragilis	0.452618301	2.88370	4.37644
Lactobacillus aviarius	0.461654577	0.29268	0.16340
Akkermansia muciniphila	0.530407851	3.96129	6.69495
Bacteroides uniformis	0.545604284	11.75615	14.19226
Bacteroides caccae	0.577072064	1.40274	0.91891
Parabacteroides distasonis	0.654598391	0.73000	0.91053
Alistipes CHKCI003	0.724143408	25.43260	23.06547
Bacteroides dorei	0.769992609	4.82055	4.17077
Bacteroides clarus	0.847346211	0.76005	0.85229
Clostridiales bacterium CHKCI006	0.995024968	0.10031	0.10058
Lactobacillus gallinarum	0.086962228	0.31513	0.06580
Lactobacillus johnsonii	0.270108965	0.11333	0.04414
Clostridium spiroforme	0.33592567	0.16040	0.08456
Bacteroides finegoldii	0.101930031	0.06458	0.32663
Gemmiger An50	0.141173219	0.03429	0.17206
Clostridium CAG 242	0.340614463	0.06019	0.23187
Corynebacterium stationis	0.377440677	0.01764	0.17336

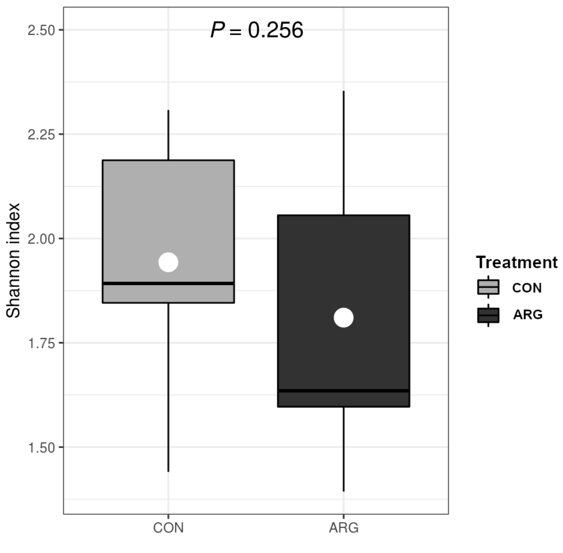

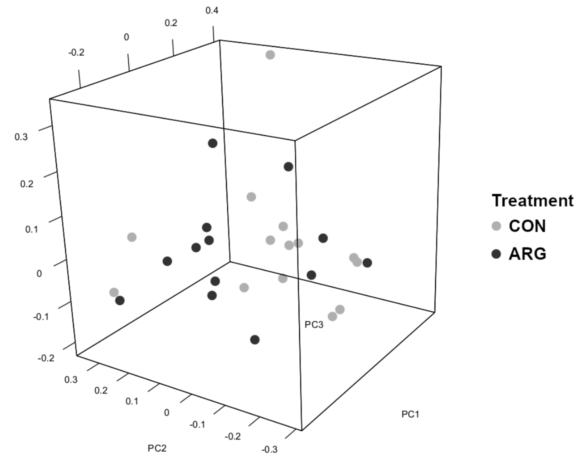

Supplement: Supplementary file 2 — Additional file 2. Relative abundances of bacteria and plots of alpha (Shannon) and beta diversity of caecal content samples of CON and ARG birds at D49. [file 40104_2023_839_MOESM2_ESM.pdf]
